# The current position of surgical lung biopsy in the diagnosis of idiopathic pulmonary fibrosis

**DOI:** 10.1186/1465-9921-14-43

**Published:** 2013-04-15

**Authors:** Riitta Kaarteenaho

**Affiliations:** 1 Unit of Medicine and Clinical Research, Pulmonary Division, University of Eastern Finland, PO Box 1777, Kuopio, 70211, Finland; 2 Center for Medicine and Clinical Research, Division of Respiratory Medicine, Kuopio University Hospital, PO Box 1777, Kuopio, 70211, Finland; 3 Respiratory Research Unit and Clinical Research Center, Oulu University Hospital, Oulu, Finland

**Keywords:** Fibroblast focus, Complication, Non-specific interstitial pneumonia, Open lung biopsy, Usual interstitial pneumonia, Video-assisted thoracoscopy

## Abstract

A new international statement defines usual interstitial pneumonia (UIP) which is a histological and radiological form of idiopathic pulmonary fibrosis (IPF) more precisely than previously. In the diagnosis of IPF, either in high resolution computed tomography (HRCT) a UIP pattern must be present or alternatively specific combinations of HRCT and surgical lung biopsy findings can be accepted. In about two third of the cases IPF can be diagnosed by clinical and radiological criteria. Thus surgical lung biopsy is needed in about one third of cases to achieve the ultimate diagnosis, which requires multidisciplinary cooperation. In large clinical trials conducted during the last decade, lung biopsy was performed in about 30–60% of the cases. The most serious complication of lung biopsy is mortality within 30 days after the procedure, with a frequency of about 3–4% reported in most studies. Because of the histological variability, surgical lung biopsy should be taken from a minimum of two lobes. The number of fibroblast foci in surgical lung biopsy has been shown to correlate with survival in several studies.

## Review

Until recently, surgical lung biopsy (SLB) has been regarded as the golden standard in the diagnosis of idiopathic pulmonary fibrosis (IPF) and other types of idiopathic interstitial pneumonias (IIP). During the past decades, the pathological and clinical terms for the IIPs have been at least partly different which has been responsible for misinterpretations between different specialists working with the patients with IIP.

The new international statement on the diagnosis and management of IPF has recommended adopting a multidisciplinary approach for the ultimate diagnosis [1]. On the other hand, when a patient has a clinically and radiological typical usual interstitial pneumonia (UIP) with no known causes, the diagnosis can be made without bronchoscopy, transbronchial biopsy (TBB), bronchoalveolar lavage (BAL) or SLB procedures. Thus the role of the traditional investigative methods including SLB is currently changing. When previously, SLB has been performed as a basic diagnostic tool for investigating a patient with suspected IPF, it is nowadays needed for the ultimate diagnosis of the non-typical IPF patients who do not fulfill the precise criteria for UIP in high-resolution computed tomography (HRCT) [2,3]. These non-typical IPF patients may have confounding aspects and other diseases, which can complicate the diagnostics. In this review article, the current histological criteria of UIP will be outlined as well the current awareness of the necessity, commonality and risks of SLB. The accuracy of histological diagnosis, the variability of the histological features and the significance of SLB as a prognostic tool are also discussed.

## Role of histological diagnostics in the 2011 international statement of IPF

In a new statement, IPF is defined as a progressive interstitial pneumonia of unknown cause, occurring in adults, limited to the lungs and associated with the histological and/or radiological pattern of usual interstitial pneumonia (UIP). In the diagnosis of IPF, other known causes must be excluded, in HRCT, a UIP pattern must be present, or specific combinations of HRCT and SLB findings can be accepted. Further, there are no longer the so-called major or minor criteria present in the previous guidelines, and BAL or transbronchial biopsy is no longer required as was the case in the statement released in 2000 [1,4].

**Figure 1:**
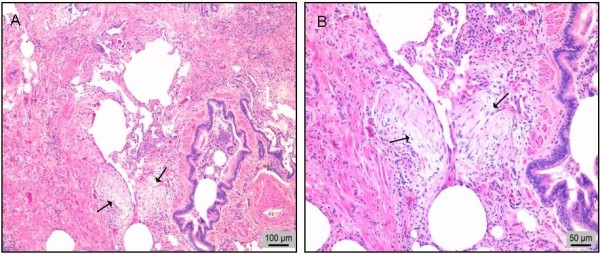
**Histopathological findings of a surgical lung biopsy sample. A**. An image showing histological features of usual interstitial pneumonia (UIP) including dense fibrosis, fibroblast foci (arrows) and only a few nearly normal looking alveolar walls (on the middle). **B**. Fibroblast foci (arrows) are seen at higher magnification. Haematoxylin-eosin (HE) stain.

The new diagnostic criteria for radiology and histopathology are more precise than previously and now include 3 HRCT categories and 4 histological categories, namely 1) UIP, 2) possible UIP and 3) inconsistent with UIP for HRTC, and 1) UIP, 2) probable UIP, 3) possible UIP and 4) not UIP for histopathology. The histological categories are presented in detail in Table 1. In the typical UIP pattern, a marked fibrosis, a patchy involvement and fibroblast foci (FF) should be present (Figure 1).

**Table 1:** **Histopathological criteria for UIP/IPF**[1]

**1. UIP pattern (All four criteria)**	**2. Probable UIP pattern**	**3. Possible UIP pattern (All three criteria)**	**4. Not UIP pattern (Any of the six criteria)**
1. Marked fibrosis/architectural distortion, ± honeycombing in a predominantly subpleural/paraseptal distribution	1. Marked fibrosis/architectural distortion, ± honeycombing	1. Patch or diffuse involvement of lung parenchyma by fibrosis, with or without interstitial inflammation	1. Hyaline membranes
			2. Organizing pneumonia
			3. Granulomas
2. Presence of patchy involvement of lung parenchyma by fibrosis	2. Absence of either patchy involvement or fibroblast foci, but not both	2. Absence of other criteria for UIP (see UIP pattern column)	4. Marked interstitial inflammatory cell infiltrate distant from honeycombing
3. Presence of fibroblast foci	3. Absence of features against a diagnosis of UIP suggesting an alternate diagnosis (see fourth column)	3. Absence of features against a diagnosis of UIP suggesting an alternate diagnosis(see fourth column)	5. Predominant airway centered changes
4. Absence of features against a diagnosis of UIP suggesting an alternate diagnosis (see fourth column)	OR		6. Other features suggestive of an alternate diagnosis
	4. Honeycomb changes only		

Abbreviations:

*IPF* idiopathic pulmonary fibrosis.

*UIP* usual interstitial pneumonia.

The 2011 statement recommends adoption of the following diagnostic algorithm for patients with suspected IPF [1]. Patients should be carefully evaluated for identifiable causes of interstitial lung disease (ILD). In the absence of an identifiable cause for ILD, an HRCT demonstrating a UIP pattern is diagnostic of IPF. In the absence of a UIP pattern, and presence of possible UIP or not UIP features in HRCT, SLB should be performed after which IPF can be diagnosed by the examining combination of HRCT and histological patterns. The accuracy of the diagnosis of IPF has been shown to be increased if one conducts multidisciplinary discussions with other ILD experts [5].

## How often SLB is performed?

The study of Raghu et al. indicated that IPF can be diagnosed by clinical and radiological criteria in about two third of all cases [6]. Subsequent studies have later confirmed that the characteristic HRCT features are absent in around 30% to 40% of IPF patients and thus, in about 1/3 of IPF patients SLB may be needed to achieve an accurate diagnosis [7-11]. Hunninghake and co-authors found that an expert panel of pathologists was more accurate in making a diagnosis of IPF than a panel of radiologists and clinicians, and moreover, that accuracy was higher among the groups of experts than that for referral centers [8]. Another study revealed that the accuracy of the diagnosis of IPF increased with clinical, radiologic, and histological correlation [5].

The most common differential diagnostic problem is nonspecific interstitial pneumonia (NSIP). Flaherty and co-authors claimed that patients with an HRCT pattern of UIP are likely to exhibit a histological pattern of UIP, but the patients with an HRCT pattern other than UIP may have either histological UIP or NSIP in the SLB specimen, and that HRCT has limited specificity in identifying histological proven NSIP [7]. Silva and others observed that in 28% of the biopsy-proven NSIP cases, the HRCT features that were originally suggestive for NSIP changed to findings that were indicative of IPF [12]. Although CT features are typical for UIP in most case, sometimes CT findings may mimic NSIP, chronic hypersensitivity pneumonitis (HP) or sarcoidosis [13]. Some studies have revealed that HRCT may permit a distinction between NSIP and IPF in about 70% of patients [14,15]. However, a recent study showed, that the characteristic HRCT features of chronic HP, IPF and NSIP allow one to make a confident differentiation between these entities in approximately 50% of the patients [16].

There are some published data about the frequency of SLB in different countries, which have shown the actual incidence of SLB in routine clinical diagnostics during the 1990’s and 2000’s. Epidemiologic studies in Italy, Belgium, Greece, Spain, United States and Finland have revealed that the diagnosis has been confirmed by SLB in 28–38% of the cases [17-22]. Questionnaire-based national surveys have shown that in Greece and Spain approximately 31% of the patients with IPF were diagnosed by SLB [19,22]. In the major clinical trials conducted in the past decade, the current diagnostic criteria for IPF have been used which may provide some enlightenment on the frequency of SLB. In the Japanese studies examining pirfenidone, about 20–30% the IPF patients were biopsied. In contrast in the Capacity studies, the numbers of SLBs were much higher, with about 40–60% of biopsied patients (Table 2) [23-25]. The percentages of the SLBs were similar in the IFIGENIA and also in the first large interferon-study [26,27]. Furthermore, a high percentage of biopsies was encountered in the studies of INSPIRE, BUILD-1, imatinib and etanercept [28-31]. In the study of triple-kinase, the percentages of SLB were lower, by approximately 30% (Table 2) [32].

**Table 2:** Number of biopsied IPF-patients in the clinical trials conducted during the past decade

**Study**	**Number of patients**	**Number (n)/% of biopsied patients**
**Pirfenidone**	107	- pirfenidone: n = 15/21%
Azuma et al., AJRCCM 2005		- placebo: n = 8/23%
**Pirfenidone**	267	- high dose: n = 26/24%
Taniguchi et al., Eur Respir J 2010		- low dose: n = 16/29%
		- placebo: n = 28/27%
**Pirfenidone**	CAPACITY I	CAPACITY I
Noble et al., Lancet 2011	435	- 1197 mg: n = 32/37%
CAPACITY I	CAPACITY II	- 2403 mg: n = 86/49%
CAPACITY II	334	- placebo: n = 85/49% CAPACITY II
		- pirfenidone: n = 94/55%
		- placebo: n = 94/54%
**Triple-therapy**	155	- triple therapy: n = 38/48%
Demedts et al., NEJM 2005		- control: n = 35/47%
IFIGENIA		- excluded patients: n =24/89%
**Interferon gamma-1b**	330	- IFN-γ-1b: 62%
Raghu et al., NEJM 2004		- placebo: 67%
**Interferon gamma-1b**	826	- IFN-γ-1b: n = 305/55%
King et al.,		- placebo n = 151/55%
Lancet 2009		
INSPIRE		
**Bosentan**	158	- bosentan: 68%
King et al.,		- placebo: 60%
AJRCCM 2008		
BUILD-1		
**Imatinib**	119	- imatinib: n = 25/42%
Daniels et al.,		- placebo: n = 29/48%
AJRCCM 2010		
**Etanercept**	88	- etanercept: n = 28/46%
Raghu et al.,		- placebo: n = 23/41%
AJRCCM 2008		
**Triple-kinase**	432	- placebo: n = 19/22%
Richeldi et al.,		- 50 mg × 1: n = 25/29%
NEJM 2011		- 50 mg × 2: n = 27/31%
		- 100 mg × 2: n = 20/23%
		- 150 mg × 2: n = 29/34%

## Comparison of video-assisted thoracoscopic operation and open lung thoracotomy for SLB

Several studies have shown that SLB taken by either open lung thoracotomy (OLB) or video-assisted thoracoscopic surgical (VATS) operation is an efficient diagnostic procedure for ILD [33-36]. Mouroux et al. compared the efficacy and safety of VATS and OLB in the diagnosis of ILD and revealed that in conjunction with compatible efficacy and similar morbidity and mortality, VATS offered several advantages such as reduction of the operative time and hospital stay [37]. In contrast, in a randomized and controlled trial, it was observed that there were no differences in outcomes for thoracoscopic and thoracotomic procedures [38]. The recent statement of IPF stated that the decision on which procedure to perform in the SLB needs to be based on individual patient characteristics and surgical expertise, and whether or not to pursue SLB should be evaluated depending on the clinical situation of the individual patient [1].

## Risks of SLB procedure

The most common complication of SLB is prolonged air-leak which occurs in about 6–12% of the cases; other common complications can be a need for mechanical ventilation, pneumonia, pneumothorax, hemothorax, pleural effusion, empyema and prolonged ventilation [39-41]. In an Icelandic study, the general complication rate was 16% and the 30-day mortality was less than 3 percent [40]. The most serious complication is mortality within 30 days after the procedure which is commonly caused by an acute exacerbation of IPF, a phenomenon that is characterized by diffuse alveolar damage (DAD) superimposed on UIP [42]. The detailed mechanisms leading to DAD after the SLB procedure are not well known. After the first days of injury DAD is characterized by hyaline membranes, edema and interstitial acute inflammation, whereas later during the organizing phase alveolar septal thickening with loose organizing fibrosis, type II pneumocyte hyperplasia, and patchy or diffuse airspace organization exist. The histological feature of DAD is known to be associated also with many other lung diseases like acute interstitial pneumonia (AIP), severe viral lung infections and acute respiratory distress syndrome (ARDS) [42].

Table 3 lists the studies which have evaluated the mortality within 30 days after the SLB procedure [39-41,43-45]. In some studies, the mortality within 90 days was also evaluated. All studies were retrospective and the number of the patients has been rather low. In addition to IPF, some other types of ILDs like NSIP and connective tissue disease associated interstitial lung diseases (CTD-ILD) have been also included in most of the studies. The study of Utz and co-authors revealed the highest mortality, nearly 17% [45]. In that particular study, in which the patients were operated during the years 1986–1995, most of the biopsies had been taken by open thoracotomy and less by video-assisted thoracoscopic operation, while in other studies only a minority of the patients had been operated by open thoracotomic surgery. In most of the studies, the mortality was lower than in that reported by Utz, namely approximately 3–4% [39,40,43,44]. In the study by Park and co-authors no difference in mortality between OLB and VATS was observed, while in another study no mortality in the patients operated by VATS was observed since all the patients who had died within 30 days had been operated by OLB [44]. The number of the SLBs in these studies has been variable. In the study of Tiitto et al. only one SLB was taken from most of the patients [44], whereas in some other studies, the number of the biopsies varied from 2 to 4 with no marked effect on the complication rate or the short term mortality [39,41]. In the abovementioned studies, the risk factors for the mortality within 30 days were the existence of acute exacerbation of IPF at time of biopsy, low diffusion capacity (DLCO), mechanical ventilation, immunologic treatment, OLB and an age of more than 67 years.

**Table 3:** Studies of mortality within 30 days after the surgical lung biopsy procedure

**Study**	**Patients**	**Type of biopsy**	**Number of biopsies/Period**	**Mortality 30 days (90 days)**	**Risk factors**
Utz et al., Eur Respir J 2001	IPF = 46	OLB 73%	No of biopsies not described	- 16.7% in total population	- 4/10 had AE-IPF before SLB
	CTD-UIP = 14	VATS 27%	1986-1995	- All IPF	- Lower DLCO
				- 21.7% in IPF	
Lettieri et al., Chest 2005	IPF = 42	OLB (no?)	No of biopsies not described	- 4.8%	- Mechanical ventilation
	Non-IPF = 41	VATS (no?)	1996-2002	(5/6%)	- Immunologic treatment
				- No difference IPF vs non-IPF, VATS vs OLB	
Tiitto et al., Chest 2005	IPF = 64	OLB n = 42	1	- OLB 5.3%	- OLB
	CTD-UIP = 12	VATS n = 34	1973-2002	- VATS 0%	
Park et al., Eur J Cardio-Thorac Surg 2007	IPF = 140	OLB n = 50	2-3	- 4%	- AE-IPF
	NSIP = 46	VATS n = 150	1990-2003	(8%)	- DLCO < 50%
	COP = 14				
	No ICU patients				
Sigurdsson et al., Ann Thorac Surg 2009	UIP = 23	OLB n = 45	1: 70%	- 3%	- No data of different ILDs
	OP = 17	VATS n = 28	2: 20%	(4%)	
	NSIP = 6		1986-2007	- No difference OLB vs VATS	
	Others = 27				
Fibla et al., Int J Cardiovasc Thorac Surg 2012	IPF = 122	OLB 10%	1: 16%	VATS 9%	- Age > 67
	COP = 31	VATS 90%	2: 64%	OLB 10.6%	- OLB
	RBILD = 16		3: 59%		- Immunologic treatment
	NSIP = 13		4: 1%		- No data of different ILDs
	Others = 114		2002-2009		
	6% in ICU				

Abbreviations:

*AE-IPF* acute exacerbation of idiopathic pulmonary fibrosis.

*COP* cryptogenic organizing pneumonia.

*CTD-ILD* connective tissue associated interstitial lung disease.

*DLCO* diffusion capacity.

*ILD* interstitial lung disease.

*ICU* intensive care unit.

*IPF* idiopathic pulmonary fibrosis.

*NSIP* nonspecific interstitial pneumonia.

*OLB* open lung thoracotomy.

*RBILD* respiratory bronchiolitis and interstitial lung disease.

*SLB* surgical lung biopsy.

*VATS* video-assisted thoracoscopic operation.

The study of Park et al. showed that SLB performed at the time of acute exacerbation of IPF resulted in higher 30-day mortality (28.6%) compared to the patients without acute exacerbation (3.0%) [39]. Utz and co-authors presented in their study that 10 of 68 patients with UIP died within 30 days after the procedure, when accelerated decline of the disease was the reason for performing SLB in 4 (40%) patients [45]. Fibla and others observed that 28 (9%) of 331 patients died within 30 days, of which 9 was in preoperative intensive care treatment [41]. That particular study revealed high rate for severe complications like acute exacerbation for respiratory failure (26.1%) and postoperative need for intensive care (22.7%).

Because of the retrospective and descriptive nature of the studies, it is difficult to draw any firm conclusions on the risk factors or limits for SLB. On the other hand, it is probable that no controlled clinical trial will be performed on this particular topic in the future. If one wishes a more accurate estimation of the risks of SLB, then larger patient material from multicenter studies may be required. A new method for performing SLB has been recently presented, such as awake thoracoscopic biopsy, which was shown to be feasible requiring only regional anesthesia and it has resulted in low morbidity, excellent diagnostic yields, short hospital stays, and low costs [46].

## Accuracy of histologic diagnosis and histologic variability of SLB

It has been shown that there exists an inter-observer variability between pathologists when analyzing SLB samples. The study of Nicholson et al. revealed that there was only fair agreement among pathologists who had been provided with very little clinical information of the patients [47]. The previous studies analyzing the inter-observer error among clinicians and radiologists have shown similar kinds of results [48,49]. The multicenter study of Thomeer et al. observed a fair agreement in histological analysis between several investigators, with those of HRCT analyses were fair to moderate [50]. In that particular study, the lung biopsy material comprised of 44 SLB and 38 transbronchial biopsy samples, which might have had some effect on the result. The accuracy of diagnosis has been shown to be higher among expert specialists than the corresponding value made by physicians in the referring centers [5].

Some studies have indicated that the histological features may be variable in some cases of IPF since in some lobes or segments of lung NSIP-like features can exist in addition of UIP features. The patients with the coexistence of both NSIP and UIP patterns seemed to behave similarly to those with only a UIP pattern [51,52]. Sampling errors may result if only one biopsy is taken since it has been shown that in 26% of the cases, the histological classification would have been different between UIP and NSIP if only one biopsy had been taken. These findings laid the foundation for the current recommendation that SLB should be taken from a minimum of two lobes, and preferably more [51].

NSIP is nowadays a recognized entity exhibiting, however, overlapping features with other types of IIPs and hypersensitivity pneumonia. Unlike IPF, the prognosis of NSIP is good and it occurs mostly in middle-aged women who are never-smokers [53]. At present, the evidence that fibrotic form of NSIP is a precursor of UIP is weak. Possibly a subset of patients mistakenly diagnosed as having fibrotic NSIP may ultimately prove to have IPF. Familial form of IIP has been often called as familial IPF. A recent study showed, however, that less than half of the patients with familial IIP had histological strictly defined UIP features, but rather more often unclassifiable fibrosis [54].

## SLB as a tool for a marker of prognosis and the significance of fibroblast foci

In addition of the diagnosis of ILD, SLB can be used also as source of a biomarker for the prognosis in IPF patients. The number of fibroblast foci (FF) i.e. active centers of fibrogenesis consisting of myofibroblasts, fibroblasts and extracellular matrix (ECM) proteins and lined by regenerative hyperplastic or metaplastic alveolar epithelium, has been shown to correlate with patient survival in several studies [47,55-59], but not in all [60] (Table 4). The detailed information of the studies focusing on the number of fibroblast foci is presented in the Table 4.

**Table 4:** Studies of the association between the numbers of fibroblast foci (FF) to the prognosis of IPF

**Study**	**Patients and method**	**Results**
King et al., Am J Respir Crit Care Med 2001	87 IPF	Granulation/connective tissue score i.e. FF was a significant predictor of survival in patients with IPF
	Semiquantitative	
	Stainings: HE, pentachrome, Prussian blue and toluidine blue	
Nicholson et al., Am J Respir Crit Care Med 2002	53 IPF	Mortality of the patients was linked to an increasing FF score, which associated also with greater declines in FVC and DLCO
	Semiquantitative	
	Staining not described	
Flaherty et al., Am J Respir Crit Care Med 2003	99 IPF and 9 with connective tissue disease (CTD-UIP)	The profusion of FF associated with the survival of UIP in whole study material, but not in IPF
	Semiquantitative	The patients with IPF had higher profusion of FF than the patients with CTD-UIP
	Staining not described	
Tiitto et al., Thorax 2006	64 IPF and 12 CTD-UIP	The number of FF correlated with the survival of the patients. The patients with ≤ 50 FF/cm^2^ had a median survival of 89 months compared with 49 months in those with >50 FF/cm^2^
	The total number of FF was counted in the area of which was defined by image analysis. The number of FF was divided into two subgroups (≤ 50 or >50 FF/cm2)	
	Stainings: AB-PAS and HE	The number of FF was lower in CTD-UIP than in IPF
Enomoto et al., Chest 2006	53 IPF	%FF score was a significant predictor of survival in IPF patients
	Images of sections were studied by image analysis. %FF was calculated by dividing the area of FF by that of the target field. Overall %FF in each patient was defined as the average %FF > 10 selected cases	
	HE staining	
Hanak et al., Respir Med 2008	43 IPF	No significant relationship between FF profusion and survival
	FF was counted by using a conventional point-counting technique. The number of points intersecting FF was expressed as a fraction of the total points counted on each slide and a mean value was calculated	
	Staining not described	
Lee et al., Sarcoidosis Vasc Diffuse Lung Dis 2011	86 IPF	FF score associated with survival
	Semiquantitive	
	Staining not described	

Abbreviations:

*AE-IPF* acute exacerbation of idiopathic pulmonary fibrosis.

*AB-PAS* Alcian blues periodic acid Schiff.

*COP* cryptogenic organizing pneumonia.

*CTD-ILD* connective tissue associated interstitial lung disease.

*DLCO* diffusion capacity.

*HE* haematoxylin-eosin.

*ILD* interstitial lung disease.

*ICU* intensive care unit.

*IPF* idiopathic pulmonary fibrosis.

*NSIP* nonspecific interstitial pneumonia.

*RBILD* respiratory bronchiolitis and interstitial lung disease.

*OLB* surgical lung biopsy taken by thoracotomic surgical operation.

*VATS* surgical lung biopsy taken bb video-assisted thoracotomic surgical operation.

Despite this quite convincing evidence, the counting of FF in biopsies has not yet become a common routine clinical practice. This might be attributable to the fact that the number of FF has been evaluated with variable methods in the different studies. At present FF has proved to be the only reproducible histological factor that correlates with the prognosis in IPF. There are very few studies, which have evaluated the value of immunohistochemical markers from SLB specimens on survival, since during the past decade most studies focusing on biomarkers have been conducted by using serum or BAL samples. Nearly two decades ago it was reported that a high expression level of the ECM protein, tenascin-C as detected by immunohistochemistry in SLB samples, correlated with shortened survival in patients with UIP [61].

## Transbronchial biopsy in the diagnosis of IPF

Neither the previous nor the current statement has recommended TBB for the diagnosis of IPF; this technique has been used mainly to exclude other ILDs such as sarcoidosis, or malignancies and infections [1,4]. The study of Berbescu et al. however showed that characteristic histologic features of UIP could be identified from TBB specimens more often than previously appreciated observing changes diagnostic of UIP in 43% of the cases [62]. Another study showed that TBB could reveal a UIP pattern in about 30% of the cases [63]. In contrast Shim and others found UIP features in only 9.4% of the patients [64]. In a recent review article it was presented that in the right clinical setting and with appropriate tissue sampling TBB can support a diagnosis of UIP fairly often being especially useful in elderly patients or those with advanced fibrosis in whom there is significant mortality and morbidity from SBL [65]. It is notable that TBB samples in the abovementioned studies have been analyzed by the expert pulmonary pathologists, which may emphasize the need for refer these cases to the specialized centers. A previous study showed that by using a novel technique for TBB, namely transbronchial cryobiopsy, the size of TBB samples were much larger than those obtained using forceps [66]. In a recent prospective study TBB cryobiopsy samples obtained from 40 patients were evaluated, of which in 85% of the cases at least two of three typical UIP features were present [67].

## Conclusion

It is probable that approximately one third of the IPF patients would need SLB in order to obtain an accurate diagnosis. Several remarkable points should be considered when making a decision whether or not to perform the SLB. The mortality, which can occur shortly after the procedure, has probably been the major reason to refrain from performing SLB. The precise risk limits for complications of SLB procedure are not well known. Thus a thorough deliberation taking into consideration age, other diseases, medication, lung function and the stage of the pulmonary fibrosis are needed before proceeding to a procedure of SLB. As it has been noted in the new statement, in patients with severe physiologic impairment or substantial comorbidity, the risks of SLB may outweigh the benefits of establishing a secure diagnosis of IPF, and moreover, clinicians must spend adequate time with patients to discuss patients’ values and preferences [1].

The accuracy of the histological diagnosis might be problematic, and thus it seems to be fundamental that SLB are analyzed by experienced pulmonary pathologists. It is probable that the histological diagnosis may become even more challenging than previously when only the patients with non-typical HRCT features are biopsied, and UIP may be at risk of remaining obscured by other concomitant diseases or confounding factors. Moreover, the challenge of pulmonary pathology will be greater because of the fact that more complex and less classical lung diseases will be investigated that will make classification of histological patterns more difficult and may limit the role of pathology as a routine predictor of prognosis. Evaluation of the pathological archives from previous decades might help in the training of pulmonary pathologists and for gathering experience from a large amount of samples including also the typical cases representing IPF/UIP. It is likely that more SLB may be needed for the diagnosis of NSIP and for the exclusion of IPF, especially after the preliminary results of the Panther-study which revealed a harmful effect of triple therapy treated IPF patients [68].

Since many IPF patients are at an older age and suffer from many other diseases, or their lung disease has already progressed to an advanced stage, SLB cannot be considered as a safe procedure for all individuals. Thus, there is a definite need for developing new diagnostic tools for the diagnosis of IPF and other ILDs. According to the current statement, BAL or TBB are no longer required for the diagnosis of IPF. A new BAL guideline has been recently published [69] as has as a new BAL cell culture technique for diagnostic samples [70]. Hopefully, BAL and TBB will gain greater acceptance in IPF diagnostics in the future with the development of more standardized techniques and innovative methodologies. Improvements in all methodological techniques including radiology and also the invasive procedures such as SLB, TBB and BAL could be predicted to make the diagnostics of IPF and other type of ILDs not only faster and safer but also more accurate, which will be important in the future due to a new classification of IIP including a category of unclassifiable ILD [71,72].

## Competing interest

The author declares that she has no competing interests.
